# Correlation between reduced hepatotoxicity and affinity of antipsychotics to specific serotonin receptors in a spontaneous reporting database

**DOI:** 10.1192/j.eurpsy.2023.680

**Published:** 2023-07-19

**Authors:** R. Zeiss, M. Gahr, S. Hafner

**Affiliations:** ^1^Department of Psychiatry, University of Ulm, Ulm; ^2^Department of Psychiatry, Hospital for Psychiatry, Psychotherapy and Psychosomatic Medicine Werneck Castle, Werneck; ^3^Institute of Pharmacology of Natural Products and Clinical Pharmacology, University of Ulm, Ulm, Germany

## Abstract

**Introduction:**

Drug-Induced Liver Injury (DILI) is one of the most common causes of hospitalization due to liver failure and represents a considerable challenge in clinical practice. One risk factor is the long-term use of a drug. Antipsychotics are regularly prescribed over a long period of time. Therefore, potential hepatotoxicity is of particular importance here. However, DILI related to antipsychotics are still insufficiently understood.

**Objectives:**

Within a combined pharmacoepidemiologic and pharmacodynamic approach, we examined the association between DILI and the receptor affinity of various common prescribed antipsychotics.

**Methods:**

Disproportionality analyses were used to calculate reporting odds ratios (RORs) for reports in which drug-related hepatic disorders were reported as an adverse reaction related to antipsychotics. MedDRA terms for Drug related hepatic disorders were used to identify cases. Data were extracted from VigiBase the WHO global database of reported potential side effects of medicinal products. For pharmacodynamic evaluation, we calculated Pearson correlation coefficients between affinity for various receptors and the corresponding RORs.

**Results:**

We observed a statistically significant (r (12) = -. 74, p = 0.002384) negative correlation between 5-hydroxytryptamine receptor 1A receptor affinity and drug related hepatic disorders. Furthermore, we observed a statistically significant (r (8) = -. 69, p = 0.02577) negative correlation between 5-Hydroxytryptamine receptor 2B receptor affinity and drug related hepatic disorders. No statistically significant association was found for other receptors.

**Conclusions:**

In this exploratory pharmacoepidemiological and pharmacodynamic approach, no particular risk for increased hepatotoxicity related to affinity for a specific receptor was found. Interestingly, a negative correlation to two serotonin receptors was found. These findings are consistent with results from the animal model, in which improved liver function and reduced fibrogenesis were observed under 5HT_2B_ antagonists.

**Disclosure of Interest:**

None Declared

